# Clinical characteristics and pachychoroid incidence in Japanese patients with neovascular age-related macular degeneration

**DOI:** 10.1038/s41598-022-08666-3

**Published:** 2022-03-16

**Authors:** Hidetaka Matsumoto, Junki Hoshino, Ryo Mukai, Kosuke Nakamura, Shoji Kishi, Hideo Akiyama

**Affiliations:** Department of Ophthalmology, Gunma University Graduate School of Medicine, 3-39-15 Showa-machi, Maebashi, Gunma 371-8511 Japan

**Keywords:** Eye diseases, Macular degeneration

## Abstract

The phenotypes of neovascular age-related macular degeneration (nAMD) are recognized as differing between Caucasian and Asian patients. Pachychoroid is thought to be more prevalent in Asians than in Caucasians, and may be involved in the development of nAMD in Asian patients. Therefore, we investigated the clinical characteristics and pachychoroid incidence in Japanese patients with nAMD. We retrospectively analyzed 385 eyes of 370 consecutive Japanese patients with treatment naïve nAMD. According to the nAMD nomenclature, type 1 macular neovascularization (MNV) was observed in 132 eyes (34.3%), polypoidal choroidal vasculopathy (PCV) in 137 (35.6%), mixed type 1 and type 2 MNV in 32 (8.3%), type 2 MNV in 43 (11.2%), and type 3 MNV in 41 (10.6%). Pachychoroid was seen in 58.3% of type 1 MNV, 75.2% of PCV, 34.4% of mixed type 1 and type 2 MNV, 14.0% of type 2 MNV, and 0% of type 3 MNV. Compared to nAMD patients without pachychoroid (188 eyes), those who had nAMD with pachychoroid (197 eyes) were significantly younger, had a higher proportion of males, greater central choroidal thickness, and a higher frequency of macular vortex vein anastomoses (all *P* < 0.001). Furthermore, drusen subtypes differed significantly between the two groups (*P* < 0.001). These results suggest that most Japanese nAMD patients might have type 1 MNV or PCV. Moreover, in approximately half of patients, nAMD might be associated with pachychoroid, and choroidal congestion may be involved in the development of MNV in these cases.

## Introduction

Age-related macular degeneration (AMD) is a leading cause of visual impairment in developed countries^1^. Though numerous studies on the pathophysiology and optimal treatment of AMD have been conducted, the terminologies have not been consistent. To facilitate comparison of these diverse studies, a new terminology for neovascular AMD (nAMD) was established by the nAMD nomenclature study group in 2020^2^. Macular neovascularization (MNV) was classified into types 1, 2 and 3, and mixed type 1 and type 2 MNV. Polypoidal choroidal vasculopathy (PCV) was categorized as a variant of type 1 MNV. Type 1 MNV refers to choroidal neovascularization (CNV) that grows into the subretinal pigment epithelium (RPE) space. Type 2 MNV refers to CNV that traverses the RPE and proliferates in the subretinal space. The definition of type 3 MNV is neovascularization that originates from the retinal circulation and grows toward the outer retina. Types 1, 2, and 3 MNV were previously referred to as occult CNV, classic CNV, and retinal angiomatous proliferation, respectively.

The phenotypes of nAMD are recognized as differing between Caucasian and Asian patients^3–6^. Drusen are more commonly observed in Caucasian than in Asian patients. On the other hand, the proportions of male and PCV patients are higher among Asians than Caucasians. These findings suggest that the mechanisms of MNV development in nAMD might differ, at least in some respects, between Asian and Caucasian patients. In 2013, the concept of pachychoroid was introduced, describing choroidal thickening associated with dilatation of outer choroidal vessels^7^. Pachychoroid is thought to be more prevalent in Asians than in Caucasians, and may be involved in the development of nAMD in Asian patients^8^. Our previous studies revealed that intervortex venous anastomosis is frequently observed in pachychoroid spectrum diseases^9–11^, and that the area of pachyvessels detected by en face optical coherence tomography (OCT) overlapped with the area of choriocapillaris filling delay detected in the early phase of indocyanine green angiography (ICGA)^12,13^. Therefore, we suggest that chronic ischemia of the choriocapillaris secondary to vortex vein congestion might be involved in the pathogenesis of pachychoroid spectrum diseases.

Soft drusen are sub-RPE deposits, which have been regarded as precursor lesions to nAMD and geographic atrophy^3^. Reticular pseudodrusen, first described in 1990^14^, and then later in 2010, were proven to be subretinal drusenoid deposits^15^. Reticular pseudodrusen are also known to be precursor lesions to nAMD and geographic atrophy^16^. Pachydrusen (pachychoroid-associated drusen) were introduced as new type of sub-RPE deposits in 2018^17^. It has been reported that choroidal thickness is significantly greater in eyes with pachydrusen than in eyes with soft drusen or reticular pseudodrusen^17,18^.

In this study, we assessed the prevalence of MNV subtypes in Japanese patients diagnosed with nAMD according to the nAMD nomenclature. Then, we investigated the pachychoroid incidence in each MNV subtype. Finally, we compared the demographic and clinical features including intervortex venous anastomosis and drusen subtype between the eyes of patients with nAMD with and without pachychoroid.

## Results

We retrospectively studied 385 eyes of 370 patients (271 eyes of 261 men; 114 eyes of 109 women) with previously untreated nAMD. The average patient age was 75.0 ± 9.9 years. The demographic and clinical characteristics of patients with nAMD are presented in Table 1. The type 1 MNV included 132 eyes of 130 patients (96 eyes of 95 men; 36 eyes of 35 women) with an average age of 72.9 ± 11.3 years (range: 45–96 years). PCV was diagnosed in 137 eyes of 132 patients (106 eyes of 102 men; 31 eyes of 30 women) with an average age of 74.8 ± 8.8 years (range: 52–94 years). Mixed type 1 and type 2 MNV was present in 32 eyes of 32 patients (25 eyes of 25 men; 7 eyes of 7 women) with an average age of 73.0 ± 9.3 years (range: 52–88 years). The type 2 MNV included 43 eyes of 43 patients (28 eyes of 28 men; 15 eyes of 15 women) with an average age of 76.5 ± 7.0 years (range: 59–91 years). Type 3 MNV was the diagnosis in 41 eyes of 35 patients (16 eyes of 13 men; 25 eyes of 22 women) with an average age of 82.3 ± 7.3 years (range: 65–94 years). Two patients showed type 1 MNV in one eye and mixed type 1 and type 2 MNV in the other eye. Of 385 eyes with nAMD, 301 (78.2%) had type 1 MNV or PCV, a variant of type 1 MNV. The average central choroidal thickness (CCT) was 259 ± 103 µm in type 1 MNV, 242 ± 101 µm in PCV, 215 ± 114 µm in mixed type 1 and type 2 MNV, 184 ± 89 µm in type 2 MNV, and 140 ± 61 µm in type 3 MNV, clearly diminishing in this order. Intervortex venous anastomosis was detected in 102 eyes (77.3%) with type 1 MNV, 125 (91.2%) with PCV, 26 (81.3%) with mixed type 1 and type 2 MNV, 26 (60.5%) with type 2 MNV, and 22 (53.7%) with type 3 MNV. As for drusen subtype, soft drusen and reticular pseudodrusen were more common in type 3 MNV. In the other MNV subtypes, pachydrusen were more common and about 30–40% of cases showed no drusen. Pachychoroid was seen in 77 eyes (58.3%) with type 1 MNV, 103 (75.2%) with PCV, 11 (34.4%) with mixed type 1 and type 2 MNV, 6 (14.0%) with type 2 MNV, but none with type 3 MNV.

**Table 1:** Demographic and clinical characteristics of patients with neovascular age-related macular degeneration.

MNV subtype	Type 1	PCV	Type 1 + 2	Type 2	Type 3	Total
No. of patients	130 (35.1%)	132 (35.7%)	32 (8.6%)	43 (11.6%)	35 (9.5%)	370
No. of eyes	132 (34.3%)	137 (35.6%)	32 (8.3%)	43 (11.2%)	41 (10.6%)	385
Age (years)	72.9 ± 11.3	74.8 ± 8.8	73.0 ± 9.3	76.5 ± 7.0	82.3 ± 7.3	75.0 ± 9.9
Male	95 (73.1%)	102 (77.3%)	25 (78.1%)	28 (65.1%)	13 (37.1%)	261 (70.5%)
Central choroidal thickness (µm)	259 ± 103	242 ± 101	215 ± 114	184 ± 89	140 ± 61	228 ± 105
Intervortex venous anastomosis ( +)	102 (77.3%)	125 (91.2%)	26 (81.3%)	26 (60.5%)	22 (53.7%)	301 (78.2%)
Drusen subtype						
Soft drusen	14	6	4	4	35	63
Reticular pseudodrusen	5	2	6	12	34	59
Pachydrusen	70	81	12	20	4	187
No drusen	55	51	14	12	1	133
Pachychoroid ( +)	77 (58.3%)	103 (75.2%)	11 (34.4%)	6 (14.0%)	0 (0%)	197 (51.2%)

MNV = macular neovascularization; PCV = polypoidal choroidal vasculopathy.

The comparison between nAMD with and without pachychoroid is presented in Table 2. nAMD without pachychoroid included 188 eyes of 181 patients (113 eyes of 109 men; 75 eyes of 72 women) with an average age of 78.2 ± 8.0 years. The average CCT was 183 ± 90 µm. Intervortex venous anastomosis was detected in 115 eyes (61.2%). Soft drusen were seen in 54 eyes, pseudodrusen in 55, and pachydrusen in 77, while 51 eyes had no drusen. On the other hand, nAMD with pachychoroid was seen in 197 eyes of 189 patients (158 eyes of 152 men; 39 eyes of 37 women) with an average age of 71.9 ± 10.5 years. The average CCT was 272 ± 100 µm. Intervortex venous anastomosis was confirmed in 186 eyes (94.4%). Soft drusen were observed in 9 eyes, pseudodrusen in 4, and pachydrusen in 110, while 82 eyes had no drusen. Compared to those who had nAMD without pachychoroid, patients who had nAMD with pachychoroid were significantly younger, the proportion of males was higher, CCTs were greater, and the frequency of intervortex venous anastomosis was higher (all *P* < 0.001). Moreover, drusen subtypes differed significantly between the two groups (*P* < 0.001).

**Table 2:** Comparison between neovascular age-related macular degeneration with and without pachychoroid.

	Pachychoroid (−)	Pachychoroid ( +)	*P* value
No. of patients	181	189	
No. of eyes	188	197	
Age (yrs)	78.2 ± 8.0	71.9 ± 10.5	< 0.001
Male	109 (60.2%)	152 (80.4%)	< 0.001
Central choroidal thickness (µm)	183 ± 90	272 ± 100	< 0.001
Intervortex venous anastomosis ( +)	115 (61.2%)	186 (94.4%)	< 0.001
Drusen subtype			
Soft drusen	54	9	< 0.001
Reticular pseudodrusen	55	4	
Pachydrusen	77	110	
No drusen	51	82	

## Discussion

We retrospectively studied 385 eyes of 370 patients with previously untreated nAMD. Type 1 MNV was observed in 132 eyes (34.3%), PCV in 137 (35.6%), mixed type 1 and type 2 MNV in 32 (8.3%), type 2 MNV in 43 (11.2%), and type 3 MNV in 41 (10.6%). Pachychoroid was seen in 58.3% of type 1 MNV, 75.2% of PCV, 34.4% of mixed type 1 and type 2 MNV, 14.0% of type 2 MNV, and 0% of type 3 MNV. Compared to those who had nAMD without pachychoroid, patients who had nAMD with pachychoroid were significantly younger, the proportion of males was higher, CCTs were greater, and the frequency of macular vortex anastomoses was higher. Moreover, drusen subtypes differed significantly between the two groups.

PCV reportedly accounts for a higher percentage of nAMD in Asian than in Caucasian patients^6^. Maruko et al. investigated the clinical characteristics of nAMD in Japanese patients in a hospital-based retrospective study. They reported in 2007 that the proportions of PCV and typical AMD among nAMD cases were 60.2% and 40.8%, respectively, including the patients with binocular nAMD^6^. However, in the current study, PCV patients accounted for 35.7% (132 of 370 cases), which was obviously a lower proportion than in the previous report. Moreover, type 1, mixed type 1 and type 2, and type 2 MNV, which were previously classified as typical AMD, together accounted for 55.4% (205 of 370 cases), i.e., a higher proportion than in the previous report. In Japan, AMD is the fourth leading cause of visual impairment^19^, and awareness of the disease has been rising. Therefore, it is possible that patients are seeing ophthalmologists earlier than in the past, leading to earlier detection of nAMD. It is also possible that nAMD is being detected at an earlier stage than before due to the widespread use of OCT and OCT angiography (OCTA). In fact, a multicenter study of Japanese patients with nAMD from 2006 to 2015 demonstrated significantly better visual acuity at initial diagnosis over time^20^, suggesting that more cases are receiving a diagnosis of nAMD before the disease has progressed. PCV is reportedly categorized as one of the pachychoroid spectrum diseases, and it has been suggested that the disease progresses in the order of central serous chorioretinopathy, pachychoroid neovasculopathy, and PCV^10,11,21^. Therefore, more cases might be referred to our university hospital before PNV progresses to PCV. PNV was originally defined as type 1 MNV accompanied by pachychoroid^22^. According to this definition, 58.3% of eyes with type 1 MNV were considered to have PNV in our study. Thus, it can be inferred that the proportion of PCV cases was lower and the proportion of type 1 MNV cases was higher herein than in the previous study. In addition, our previous studies have shown that PNV is characterized by a significantly thicker choroid than PCV (PNV with polypoidal lesions)^10,11^. This may be the reason for type 1 MNV showing the same choroidal thickness as PCV, even though the incidence of pachychoroid was lower than that of PCV.

It has been suggested that pachychoroid is involved in the development of nAMD in Asians, and various studies have examined this issue in recent years^8^. However, there is currently no established definition of pachychoroid, and eligibility criteria vary from study to study^23^. Recently, dilatation of the outer choroidal vessels accompanied by thinning of Sattler’s layer and the choriocapillaris have come to be considered the most important features of pachychoroid^24^. If these findings are present, pachychoroid is often diagnosed even if there is no obvious choroidal thickening. Therefore, in this study, pachychoroid was diagnosed if B-mode OCT showed dilatation of outer choroidal vessels accompanied by thinning of Sattler’s layer and the choriocapillaris, as well as the presence of MNV directly above them. Thus, pachychoroid was seen in 51.2% of eyes with nAMD in our study. Miyake et al. reported in 2015 that one fourth of Japanese cases had previously been diagnosed as having nAMD associated with pachychoroid^25^. In their study, subfoveal choroidal thickness and the absence of drusen were also included in the eligibility criteria, which may have lowered the incidence of pachychoroid in Japanese nAMD patients as compared to that in our study. However, they recently reported that 46.2% of Japanese nAMD patients showed pachychoroid-related features using deep phenotype unsupervised machine learning^26^, observations consistent with our results. Our findings suggest that pachychoroid may play a significant role in the development of nAMD in Japanese. Moreover, pachychoroid spectrum diseases have been found to be more prevalent in males^10,11^, which may account for the high percentage of males among Japanese nAMD patients.

In this study, intervortex venous anastomosis was detected at a significantly higher rate in nAMD with pachychoroid (94.4%) than in nAMD without pachychoroid (61.2%). Our prior study revealed that 40–50% of normal eyes showed intervortex venous anastomosis using en face OCT images^27^. Therefore, there may have been tiny anastomotic vessels between the superior and inferior vortex veins prior to the routine detection of any abnormalities. If the vortex veins are congested, the tiny anastomotic vessels might be dilated and easily recognized as intervortex venous anastomosis on en face OCT images. In the current study, intervortex venous anastomosis was frequently detected in nAMD with pachychoroid. However, 61.2% of eyes with nAMD without pachychoroid showed intervortex venous anastomosis, i.e., this finding was more common than in normal eyes. Therefore, mild congestion of vortex veins might be involved in the formation of intervortex venous anastomosis in nAMD without pachychoroid. In our previous study, we demonstrated pachydrusen to frequently be localized within the choriocapillaris filling delay in eyes with CSC^28^. Therefore, we speculate that circulation impairment in the choriocapillaris due to choroidal congestion might induce the development of pachydrusen. In the present study, 77 of 188 eyes (41.0%) without pachychoroid showed pachydrusen. These results also support the possibility that mild congestion of vortex veins may exist in some eyes with nAMD without pachychoroid.

In recent years, MNV has often been divided into drusen-driven and pachychoroid-driven based on the differences in their developmental mechanisms^8,21^. In the current study, 197 of 385 (51.2%) eyes with nAMD had associated pachychoroid. However, soft drusen and/or reticular pseudodrusen were also seen in 10 of these 197 (5.1%) eyes with pachychoroid. These cases may have a condition representing an overlap between drusen-driven and pachychoroid-driven MNV. On the other hand, eyes without pachychoroid were considered to have drusen-driven MNV, though, in fact, only 59 of 188 (31.9%) eyes without pachychoroid showed soft drusen and/or reticular pseudodrusen. Therefore, not all eyes without pachychoroid have drusen-driven MNV, which means there are idiopathic MNV cases that are neither pachychoroid-driven nor drusen-driven. However, the choroid is a tissue with high plasticity^29^. Even in eyes with pachychoroid, remodeling of the choroidal drainage route may reduce dilatation of the outer choroidal vessels over time, resulting in an OCT image that looks as if there is no pachychoroid. It is also possible that there are cases of MNV due to chronic ischemia of the choriocapillaris, although the vortex vein congestion is mild and there is no obvious dilatation of the outer choroidal vessels on OCT images. Such cases may, in this study, have been included among those without pachychoroid, and neither soft nor reticular pseudodrusen.

The limitations of this study include that it was single-center and retrospective, some cases with poor quality images were excluded, CCT was measured manually, pachychoroid and intervortex venous anastomosis were determined subjectively, OCT focused on the posterior pole of the fundus demonstrates only the posterior portion of the choroidal circulation, and all cases were Japanese such that the results may not be generalizable to other racial or ethnic groups.

In conclusion, most Japanese nAMD patients had type 1 MNV or PCV. About half of nAMD patients might have associated pachychoroid, and choroidal congestion may be involved in the development of MNV in these cases.

## Methods

We performed this study, in compliance with the Declaration of Helsinki guidelines, after obtaining approval from the Institutional Review Board of Gunma University Hospital. Informed consent was obtained from all individual participants included in the present study. We retrospectively studied consecutive patients diagnosed with treatment naïve nAMD, followed clinically from January 2018 through December 2020 at Gunma University Hospital. We excluded patients with low quality multimodal imaging, under −6.0 diopter of refractive error in the phakic eye, or posterior staphyloma in the pseudophakic eye.

According to our previous studies^9–11,13,30^, all patients underwent a complete ophthalmological examination, including slit-lamp biomicroscopy with a noncontact fundus lens (SuperField lens; Volk Optical Inc., Mentor, OH), color fundus photography and fundus autofluorescence (Canon CX-1; Canon, Tokyo, Japan), fluorescein angiography and ICGA with an angle of 30 degrees (Spectralis HRA + OCT; Heidelberg Engineering, Heidelberg, Germany), as well as swept-source OCT (DRI OCT-1 Triton; Topcon Corp, Tokyo, Japan, and PLEX Elite 9000; Carl Zeiss Meditec, Dublin, CA, USA). The DRI OCT-1 Triton and PLEX Elite 9000 devices incorporate a tunable laser with a 1050 nm central wavelength and acquire 100,000 A-scans/second. The DRI OCT-1 Triton has an axial resolution of 8 μm and a lateral resolution of 20 μm, while the PLEX Elite 9000 has an axial resolution of 6.5 μm and a lateral resolution of 20 μm. We obtained B-mode images of the horizontal and vertical line scans (12 mm) and radial scans (9 mm) through the fovea employing the DRI OCT-1 Triton. Next, cube data were obtained with a raster scan protocol of 1024 (horizontal) × 1024 (vertical) B-scans, which covered the 12 × 12 mm area centered on the fovea by the PLEX Elite 9000. En face images were obtained from the vitreous to the choroidoscleral border with coronal slices from a 3-dimensional dataset included in the inner software. Then, we performed OCTA volume scanning, i.e., 300 × 300 pixels in the 3 × 3 mm area demonstrated by the PLEX Elite 9000. The OCTA thus performed was based on an optical microangiography algorithm.

Following the nAMD nomenclature^2^, we classified nAMD according to MNV subtypes: Type 1 MNV, PCV, mixed type 1 and type 2 MNV, type 2 MNV, and type 3 MNV. Then, we investigated the pachychoroid incidence in each MNV subtype. In the current study, pachychoroid was diagnosed if B-mode OCT showed dilatation of the outer choroidal vessels accompanied by thinning of Sattler’s layer and the choriocapillaris, as well as the presence of MNV directly above them. Finally, we categorized nAMD into two groups, with and without pachychoroid, and compared the demographic and clinical features including age, gender, CCT, incidence of intervortex venous anastomosis, and drusen subtype between the two groups. CCT was measured on B-mode images using the computer-based caliper measurement tool included in the OCT system. CCT was defined as the distance between Bruch’s membrane and the margin of the choroid and sclera under the fovea. Intervortex venous anastomosis was considered to be present if anastomotic vessels connected the superior and inferior vortex veins on en face OCT images. The anastomotic vessels did not show narrowing toward the watershed zone^9–11^. En face OCT images at a depth of every 8 µm in the choroid were assessed. Drusen subtype was determined using fundus photographs according to previous studies^15,17^. If multiple drusen subtypes existed in the eye, they were counted in duplicate. The presence of pachyvessels on B-mode OCT images and anastomosis between the superior and inferior vortex veins on en face OCT images as well as drusen type were judged by two experienced retinal specialists (H. Matsumoto and J. Hoshino), working together.

For statistical analyses, the Mann–Whitney U test was used to compare unpaired values of age and CCT. The chi-squared independence test was used to determine differences in gender, the incidence of vortex vein anastomosis, and drusen type. The data analyses were performed using Excel (Microsoft, Redmond, WA, USA) with add-in software Statcel4^31^. A *P* < 0.05 was considered to indicate a statistically significant difference. Age and CCT are presented as the average ± standard deviation.
